# Cross-protective efficacy of NA-based mRNA vaccine candidates against seasonal and avian influenza viruses

**DOI:** 10.3389/fmicb.2026.1791088

**Published:** 2026-04-09

**Authors:** Hyunbeen Kim, Sangyi Lee, Seunghye Cho, Woojin Shin, Soyoung Lee, Sejik Park, Atanas V. Demirev, Taeyoung Lee, You-Jin Kim, Dokeun Kim, Sae Am Song, Kyung Ran Jun, Jin Il Kim

**Affiliations:** 1 Department of Microbiology, Institute for Viral Diseases, Korea University College of Medicine, Seoul, Republic of Korea; 2 Division of Infectious Diseases Vaccine Research, Center for Vaccine Research, National Institute of Infectious Diseases, Korea National Institute of Health, Osong, Republic of Korea; 3 Department of Lab Medicine, Haeundae Paik Hospital, Inje University College of Medicine, Busan, Republic of Korea; 4 Vaccine Innovation Center, Korea University College of Medicine, Seoul, Republic of Korea; 5 Biosafety Center, Korea University College of Medicine, Seoul, Republic of Korea

**Keywords:** hemagglutinin, influenza, mRNA vaccine, neuraminidase, viral variant

## Abstract

Influenza A viruses continuously evolve through antigenic drift and shift, reducing the effectiveness of vaccines that rely primarily on hemagglutinin (HA). Neuraminidase (NA), a surface antigen with greater sequence conservation, has gained attention as a complementary target for broader influenza vaccine design. Using computational analyses of 707 post-2009 A(H1N1)pdm09 NA sequences, we designed two NA-based mRNA vaccine constructs: NA-D1, derived from contemporary H1N1 isolates, and NA-E2, incorporating conserved features shared between H1N1 and H5N1. Mice received prime-boost immunization followed by homologous H1N1 or heterologous H5N1 challenge. Both NA-D1 and NA-E2 induced NA-specific antibody titers and conferred complete protection against homologous H1N1 infection. In contrast, protection against heterologous H5N1 was partial, consistent with lower predicted antigenic similarity between the vaccine constructs and H5N1 NA. Together, these findings demonstrate that NA-based mRNA vaccination can elicit robust homologous protection but offers limited heterologous protection efficacy. Our results support NA as an important complementary antigen for next-generation influenza vaccines and highlight the potential of computationally guided, dual-antigen (HA + NA) strategies to advance the development of broadly protective mRNA vaccines.

## 1 Introduction

Seasonal influenza contributes to impose a substantial global health burden, causing significant morbidity and mortality despite widespread vaccination (Iuliano et al., 2018). The antigenic diversity and rapid evolution of influenza A viruses-driven by continuous antigenic drift and occasional antigenic shift-undermine the effectiveness of vaccines that primarily focus on hemagglutinin (HA). Although vaccines are updated annually, the rapid pace of viral antigenic change and the inherent limitations of current vaccine production systems frequently lead to suboptimal strain matching and reduced vaccine effectiveness.

Influenza A viruses contain eight segmented RNA genes—polymerase basic 1 and 2 (PB1 and PB2), polymerase acidic (PA), HA, nucleoprotein (NP), neuraminidase (NA), matrix protein (M), and non-structural protein (NS) (Bouvier and Palese, 2008). HA and NA are the two major surface glycoproteins, responsible for mediating viral entry and release, respectively (Bouvier and Palese, 2008). HA, the primary target of most licensed vaccines, undergoes frequent mutations that promote immune evasion and contribute to reduce vaccine effectiveness (Bliss et al., 2024; Wang et al., 2022). In contrast, NA evolves more slowly and exhibits a higher degree of conservation, while facilitating the release of progeny virions, making it an attractive target for broader and more durable protection (Gravel et al., 2010; Leonard et al., 2024; Skarlupka et al., 2021). Accumulating evidence indicates NA-specific immunity has been shown to reduce disease severity, limit viral shedding, and decrease transmission, providing a degree of cross-protection against antigenically diverse influenza strains (Eichelberger et al., 2018; Leonard et al., 2024; Skarlupka et al., 2021).

Conventional seasonal influenza vaccines predominantly target HA, and their effectiveness is frequently compromised by antigenic mismatch (Nachbagauer et al., 2021). In addition, egg-based vaccine production is time-consuming and can introduce adaptive mutations that further reduce antigenic fidelity and limit vaccine performance (Choi et al., 2024; Estrada and Schultz-Cherry, 2019; Wang et al., 1989; Xie et al., 2015). The COVID-19 pandemic accelerated the development and global deployment of mRNA vaccine platforms, which enable rapid manufacturing, antigenic precision, and flexible adaptation to emerging viral strains (Anderson et al., 2025; Leonard et al., 2024). Although most influenza mRNA vaccine efforts have focused on HA, comparatively few studies have evaluated NA as a primary immunogen (Arevalo et al., 2022; Li et al., 2023; Tian et al., 2024), despite its conserved nature and demonstrated potential to confer cross-protection (Leonard et al., 2024; Skarlupka et al., 2021).

NA-targeted antibodies inhibit neuraminidase enzymatic activity, restrict viral spread, and complement HA-specific immune responses. A dual-antigen strategy that incorporates both HA and NA has the potential to mitigate antigenic mismatch and improve overall vaccine-mediated protection. Advances in computational biology further enable the precise identification of conserved NA residues and relevant epitopes, facilitating rational antigen design and enhancing the immunogenicity of NA-based vaccine candidates (Anderson et al., 2025).

In this study, we applied computational and phylogenetic analyses to characterize the evolutionary dynamics and structural features of NA, guiding the rational design of NA-based mRNA vaccine candidates. We then evaluated their immunogenicity and protective efficacy *in vivo.* Our findings demonstrate that NA-based mRNA vaccines induce robust homologous protection and partial cross-protection against heterologous strains. Collectively, these results support the feasibility of computationally guided NA-targeted vaccines and highlight their potential contribution to the development of broadly protective, next-generation influenza immunization strategies.

## 2 Materials and methods

### 2.1 Sequence dataset and evolutionary analysis

A total of 4,849 complete N1 sequences from human H1N1 strains collected between 2010 and 2023 were retrieved from the National Center for Biotechnology Information (NCBI)^1^ and the Global Initiative on Sharing Avian Influenza Data (GISAID)^2^ (Shu and McCauley, 2017). To reduce sampling bias, random subsampling was performed to generate three independent datasets, each comprising 704 NA sequences. These datasets were analyzed together with three reference vaccine strains: A/Brisbane/02/2018, A/Victoria/2570/2019, and A/Victoria/4987/2022. Sequence alignments were generated using Multiple Alignment using Fast Fourier Transform (MAFFT) (v7.419, RIMD, Japan) (Katoh and Standley, 2013). Evolutionary inferences were conducted using a time-framed Bayesian inference approach implemented in Bayesian Evolutionary Analysis by Sampling Trees (BEAST) (v1.10.4, BEAST Developers) (Suchard et al., 2018). The general time-reversible substitution model with invariant sites and a gamma distribution (GTR + I + G) was applied in combination with a lognormal uncorrelated relaxed clock model. The Markov Chain Monte Carlo (MCMC) chain length was initiated with 200 million runs, with sampling every 200,000 steps. Convergence was evaluated using Tracer (v1.7.1, BEAST Developers), and Maximum Clade Credibility (MCC) trees were constructed using TreeAnnotator (v 1.10.5, BEAST Developers) and visualized in FigTree (v1.4.4). To evaluate coordinated evolutionary relationship between HA and NA, tanglegram analyses was performed to identify putative reassortment events. Site-specific selective pressure was analyzed by calculating the ratio of non-synonymous to synonymous substitutions (d*N*/d*S*) per site using Datamonkey web server. A d*N/*d*S* ratio < 1 indicated negative or purifying selection, whereas a ratio > 1 suggested positive selection. The individual sites under selection were identified using fast unconstrained Bayesian approximation (FUBAR) and mixed-effects model of evolution (MEME), with thresholds applied at a posterior probability ≥ 0.9 for FUBAR and a *p*-value ≤ 0.1 for MEME. In addition, a total of 268 neuraminidase (N1) sequences derived from mammalian H5N1 isolates collected between 2022 and 2024 were retrieved from NCBI and GISAID databases to inform antigen design. Redundant sequences were removed using CD-HIT with a 99.8% sequence identity threshold to retain representative genetic diversity (Li and Godzik, 2006). The resulting curated dataset was used to define lineage-associated substitutions incorporated into the NA-E2 construct. These H5N1-derived sequences were analyzed independently from the H1N1 datasets to avoid cross-lineage bias in antigen selection.

### 2.2 Structure-based analysis of NA

To evaluate structural consequences of sequence diversity, three-dimensional NA structures were generated using SWISS-MODEL. Substitutions detected from evolutionary analyses were mapped onto the NA surface using PyMOL Molecular Graphics System (v2.5.5), enabling visualization of mutation-driven surface conformational changes (Kim et al., 2018; Mortazavi et al., 2023). These structure-based analysis informed antigen design and provided insight into the accessibility of putative epitopes.

### 2.3 mRNA preparation

HA and NA mRNA constructs were derived from A/Victoria/4987/2022 (H1N1) and codon-optimized to improve translational efficiency (Stachyra et al., 2016). The constructs were synthesized by GenScript (Piscataway, NJ) and cloned into a DNA template plasmid. mRNA was incorporated N1-methylpseudouridine instead of uridine and synthesized via *in vitro* transcription following plasmid linearization, yielding transcripts with 100-nucleotide poly (A) and Cap1 structures (Henderson et al., 2021; Kauffman et al., 2016). Purified mRNAs were encapsulated in lipid nanoparticles (LNPs) at a concentration of 1 mg/mL and stored at −80°C until use. Final LNP formulations exhibited an encapsulation efficiency > 85%, a polydispersity index < 0.2, pH 7.4 ± 0.5, endotoxin levels < 4EU/mL, and a zeta potential ± 15 mV. All doses were diluted in cold phosphate-buffered saline (PBS) immediately prior to administration.

### 2.4 Cells, viruses, and immunofluorescence expression analysis

Human embryonic kidney 293T (HEK293T; ATCC) cells and Madin-Darby canine kidney (MDCK; ATCC) cells were maintained in Dulbecco’s modified Eagle’s medium (DMEM; Gibco) or minimum essential medium (MEM), respectively, supplemented with 10% fetal bovine serum (FBS) and 1% penicillin-streptomycin. Cells were incubated at 37°C in a humidified atmosphere containing 5% CO_2_. To assess mRNA expression, HEK293T cells (3 × 10^4^ cells per well) were transfected with 200 ng of mRNA using Lipofectamine 3,000 (Invitrogen) in Opti-MEM (Gibco), as previously described (Liu et al., 2025; Zhou et al., 2024). After 24 h, cells were fixed with 4% paraformaldehyde and permeabilized. HA and NA protein expression was detected using NIBSC reference antisera (21/120 and 23/208), followed by incubation with Alexa Fluor 488-conjugated rabbit anti-sheep IgG H + L secondary antibodies (Jackson ImmunoResearch) for 1 h at room temperature (RT) in the dark. Nuclei were counterstained with DAPI solution and visualized using the Operetta Imaging System. Challenge experiments were conducted under ABSL-2 conditions using a mouse-adapted A/Korea/01/2009 (H1N1) strain and a low pathogenic avian influenza virus, A/chicken/Iksan/01/2006 (H5N1). For serological analyses, additional H1N1 strains (A/Victoria/2570/2019, A/Victoria/4897/2022) were included.

### 2.5 Mouse experiments

Female 3-week-old C57BL/6 mice were obtained from Koatech Co., Ltd. (Pyeongtaek, Republic of Korea) and housed under sterile conditions at Korea University College of Medicine. 4–6-week-old mice were vaccinated with 10 μg of mRNA encoding each antigen individually or in combination (20 μg total for bivalent vaccination) (Supplementary Figure 1). Vaccinations were administered into in the left or right hindlimb on days 0 and 14. Positive control received 10 μg of an inactivated influenza vaccine, and negative control groups received 100 μL of PBS. Serum samples were collected on day 27 (pre-challenge). On day 28, mice were challenged intranasally with 5 MLD_50_ of either mouse-adapted A/Korea/01/2009 (H1N1) or A/chicken/Iksan/01/2006 (H5N1) (Choi et al., 2014; Liu et al., 2021; Wilson et al., 2016). Following the challenge, mice were monitored daily for 14 days. Humane endpoints were defined as a ≥ 30% body weight loss (Brochut et al., 2024; Zhang et al., 2020).

### 2.6 Viral load quantification

Lungs were harvested on days 3 and 6 post-infection and homogenized in 1 mL of DMEM supplemented with 0.3% bovine serum albumin. The homogenates were clarified by centrifugation, and supernatants were collected for downstream analyses. Viral RNA was extracted from the supernatants and quantified using a one-step qRT-PCR technique (Applied Biosystems). Primers and probes were designed to target the matrix (M) protein. Cycling conditions were as follows: 48°C for 15 min, 95°C for 10 min, followed by 45 cycles of 95°C for 15 s and 60°C for 1 min. Viral RNA copy numbers were calculated from a standard curve. Infectious viral titers were determined by plaque assays in MDCK cells. Samples were serially diluted 10-fold and incubated with the cells for 60 min at RT. After removal of the inoculum, overlay medium containing 2% agarose was added. The MDCK cells were then incubated at 37°C with 5% CO_2_ for 72 h, after which overlays were removed and cells were stained with crystal violet to visualize plaques.

### 2.7 Neuraminidase inhibition assay

NAI assay was performed to quantify the inhibitory activity of NA-specific antibodies using an enzyme-linked lectin assay (ELLA), as previously established (Couzens et al., 2014). A clear flat-bottom 96-well plates were coated with fetuin (25 μg/mL; Sigma Aldrich) and incubated overnight at 4°C. Serum samples were treated with receptor destroying enzyme (RDE) and heat-inactivated at 56°C for 18 h. Two-fold serial dilutions of sera were incubated with virus adjusted to achieve 90–95% of the maximum signal within the linear region. Virus-serum mixtures were transferred to fetuin-coated plates and incubated for at least 16 h at 37°C. The plates were washed three times with PBS containing Tween-20 (PBS-T), followed by incubation with 100 μL of horseradish peroxidase-conjugated peanut agglutinin (PNA; Sigma Aldrich) for 2 h at RT. After three additional washes, O-phenylenediamine dihydrochloride (OPD; Sigma Aldrich) substrate was added and allowed to react for 1 h at RT. The reaction was stopped with sulfuric acid, and absorbance was measured at 490 nm. Percent inhibition was calculated using the following equation: 100-[(OD490_*sample*_-OD490_*dilunet*_)/(OD490_*virus*_-OD490_*diluent*_) × 100].

### 2.8 Statistical analysis

Viral RNA levels and infectious viral titers were analyzed using an unpaired two-tailed Student’s *t*-test. A *p-*value of < 0.05 was considered statistically significant. All statistical analyses were performed using GraphPad Prism version 10 (GraphPad Software, San Diego, CA).

## 3 Results

### 3.1 Phylogenetic and mutations analysis of H1N1 NA gene sequences

**FIGURE 1:**
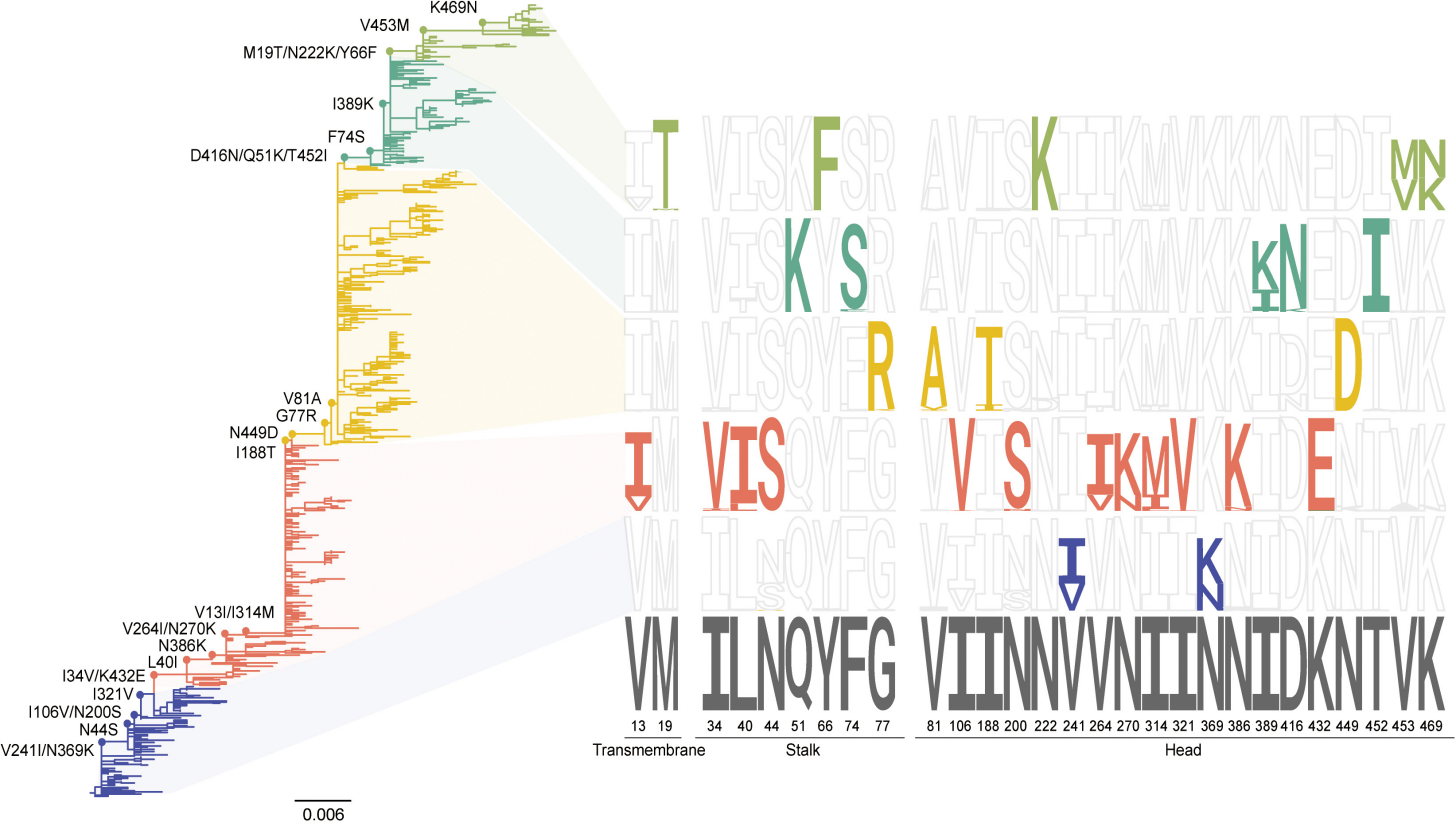
Phylogenetic analysis of NA genes from human H1N1 influenza viruses. The MCC tree of H1N1 NA genes inferred using a Bayesian evolutionary framework. Phylogenetic branches are color-coded according to lineage classification: blue, Group A; red, Group B; yellow, Group C; blue-green, Group D; and light green, Group E. The adjacent sequence logo illustrates amino acid variation across NA positions relative to the A/Korea/01/2009 (H1N1) reference. Colors highlight sites where specific amino acid residues have increased in frequency over time, indicating lineage-associated substitutions.

A total of 707 post-2009 A(H1N1) pdm09 NA sequences was analyzed and classified into five distinct groups (A-E) based on Bayesian phylogenetic analysis using BEAST (v1.10.4). The maximum clade credibility (MCC) tree revealed group-specific amino acid substitution patterns, several of which became fixed within each group (Figure 1). Sequence logo analysis demonstrates predominant mutations and reflected their temporal dynamics, consistent with the average isolation year of each group (Table 1). The dataset included three WHO-recommended vaccine strains: A/Brisbane/02/2018, A/Victoria/2570/2019, and A/Victoria/4987/2022.

**TABLE 1:** Reassortment events between NA and HA phylogenetic lineages in H1N1 influenza viruses.

Subset	Lineage pair (NA-HA)	No. of reassorted seqs	Total NA seqs	Frequency (%)
1	A-B	2	102	2.0
	B-A	1	212	0.5
	C-D	1	229	0.4
	D-C	12	107	11.2
2	D-C	11	94	11.7
	D-E	1	94	1.0
	E-D	1	50	2.0
3	C-D	1	253	0.4
	D-C	10	86	11.6

Subsets 1–3 represent independently generated datasets obtained through random sampling of H1N1 sequences. Lineage pairs (NA-HA) were defined based on phylogenetic clustering. “No. of reassorted sequences” indicates isolates where the HA and NA genes originated from different lineages. “Total NA sequences” refers to the number of NA sequences analyzed for each lineage within each subset. Reassortment frequency (%) was calculated as (no. of reassorted sequences ÷ total NA sequences) x 100.

Group A accounted for 102 of 707 sequences (14.42%) and was characterized by six amino acid substitutions: N44S, I106V, N200S, V241I, I321V, and N369K (based on N1 numbering). Among these, V241I and N369K became dominant and persisted across subsequent subsets, whereas the remaining substitutions were later observed in later-emerging group. Group B comprised 212 (29.99%) and displayed the largest number of group-defining mutations—V13I, I34V, L40I, V264I, N270K, I314M, N386K, and K432E. Several substitutions frequently co-occurred (e.g., I34V-K432E, V264I-N270K, V13I-I314M), suggesting coordinated mutational dynamics within this group. Group C was the largest group (229 sequences, 32.39%) and was defined by G77R, V81A, I188T, and N449D, which were consistently maintained across all temporal subsets. Group D consisted of 107 sequences (15.13%) and contained five characteristic substitutions (Q51K, F74S, I389K, D416N, and T452I), with Q51K and T452I frequently appeared together. Group E, the smallest group with 57 sequences (8.06%), represented the most recent isolates and include the vaccine strain, A/Victoria/4987/2022. This group was defined by M19T, Y66F, N222K, V453M, and K469N. Although M19T and N222K were not dominant across the full dataset, their presence in recent isolates underscores their potential evolutionary relevance in circulating strains.

### 3.2 Reassortment analysis of HA and NA genes

To assess reassortment between HA and NA genes, tanglegram analyses were performed comparing H1 HA and N1 NA phylogenies (Supplementary Figure 2). Most viruses exhibited concordant HA-NA pairing, with reassortment events detected at low frequencies. Reassortments was predominantly observed between Groups C and D, whereas no reassortment events were detected within Group E (Table 1).

In subset 1, Group A NA reassorted with Group B HA in 2 of 102 sequences (2.0%), and one sequence from Group B NA paired with Group A HA (1/212, 0.5%). Additional reassortment events included a single sequence from Group C NA and Group D HA (1/229, 0.4%), and twelve sequences from Group D NA reassorting with Group C HA (12/107, 11.2%). Similar patterns were observed in subset 2 and 3. In subset 2, 11 Group D NA sequences paired with Group C HA (11/94, 11.7%) and one sequence paired with Group E HA (1/94, 1%). Additionally, one sequence from Group E NA reassorted with Group D HA (1/50, 2%). In subset 3, reassortment again occurred exclusively between Groups C and D. One sequence from Group C NA paired with Group D HA (1/253, 0.4%), whereas ten Group D NA sequences reassorted with Group C HA (10/86, 11.6%). Collectively, these results indicate that HA-NA reassortment among post-2009 H1N1 viruses was infrequent and exhibited group-specific constraints.

### 3.3 Structural and evolutionary insights into H1N1 NA for vaccine design

The NA protein is categorized into two structural groups based on the presence of the 150-cavity, with N1 subtypes classified as group 1 proteins (McAuley et al., 2019). The catalytic site is composed of highly conserved residues (R118, D151, R152, R224, E276, R292, R371, and Y406), while surrounding framework residues (E119, R156, W178, S179, D198, I222, E227, E277, N293, and E425) ensure proper structural stabilization and catalytic activity (McAuley et al., 2019). Given their functional importance, mutations occurring in or near these regions have the potential to alter enzymatic properties and immunogenicity-an aspect critical for antigen design in NA-based vaccines.

**FIGURE 2:**
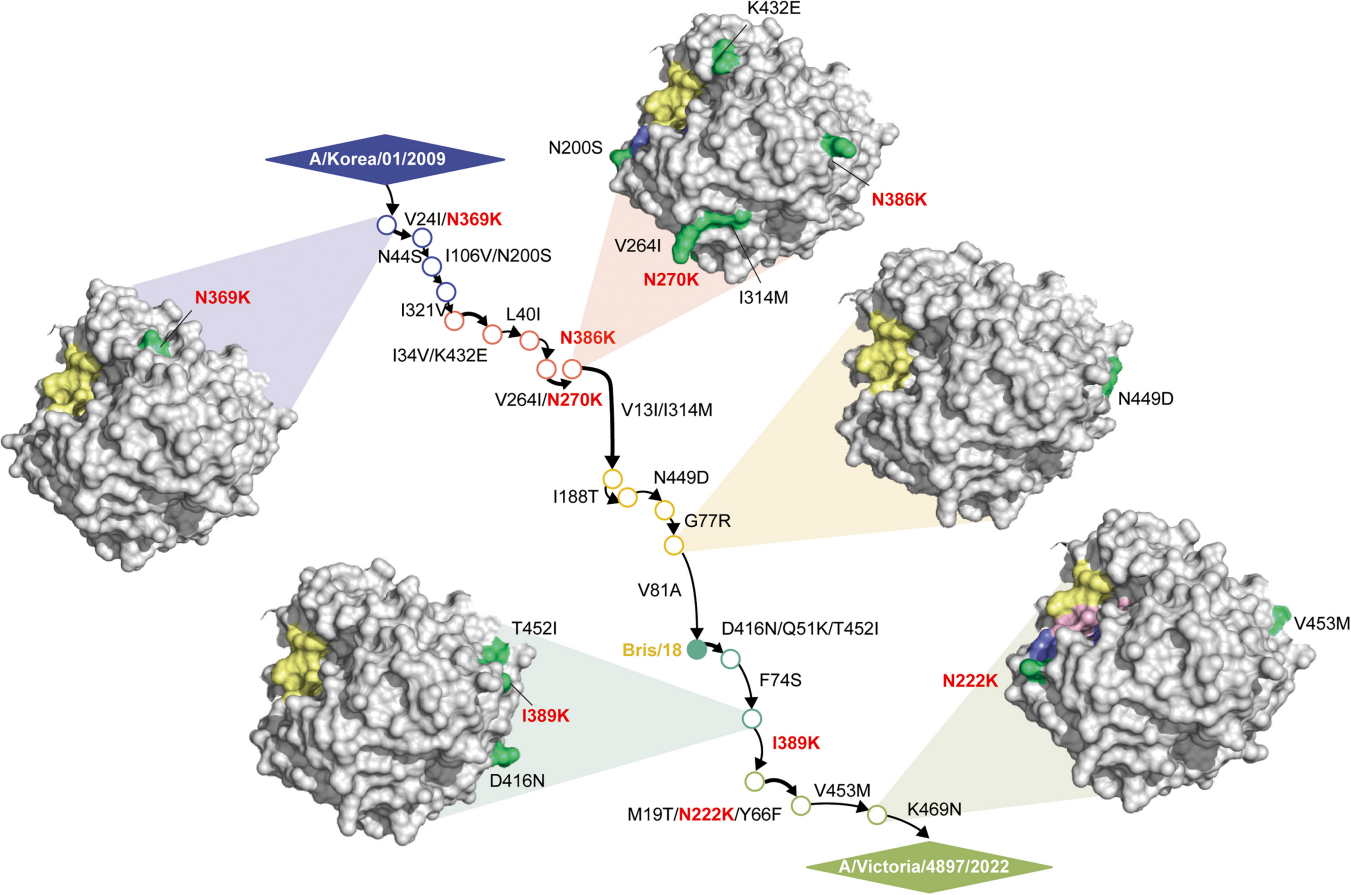
Structural visualization of key NA features. Three-dimensional representation of the NA protein highlighting major structural elements, including the 150-cavity (yellow), framework residues (purple), and the conserved enzymatic site (pink). Amino acid substitutions identified in this study are shown in green, with lysine (K) substitutions further emphasized in bold red due to their potential influence on local electrostatic properties. Arrow lengths reflect the phylogenetic distances among the corresponding NA gene lineages.

Within the NA head domain, 19 amino acid substitutions were identified, including N200S and N222K positioned adjacent to the framework region (Figure 2). Structural modeling indicated that N222K mutation interacts with D199 and I223, suggesting a role in modulating local conformational stability. Several lysine substitutions were also structurally importance. Lysine’s positively charged and outward-facing side chain enables surface electrostatic interactions; thus, substitutions such as N386K not only introduce an additional positive charge but also eliminate a glycosylation site, with potential implications for antigenicity, stability, and vaccine-induced antibody recognition.

Selective pressure analyses using MEME (*p* ≤ 0.1) and FUBAR (posterior probability ≥ 0.9) identified 24 positively selected residues (Table 2). 11 residues overlapped with recurrent mutation sites (residues 34, 74, 188, 200, 264, 270, 386, 389, 416, 452, and 469). After excluding stalk-associated residues, nine positively selected sites retained within the head domain. Residues 200 and 452 were consistently supported by both MEME and FUBAR, indicating strong evolutionary constraints at these positions.

**TABLE 2:** Positively selected sites in NA gene lineages (A–E) and in the entire dataset identified by MEME and FUBAR analyses.

Lineage	MEME	FUBAR
A	**188**	386, 469
B	74, **452**	74, **200**, 264, 416, **452**
C	**188**	34, **188**
D	−	**200**, 386, 389
E	−	270
Entire dataset	74, **200, 452**	34, **200, 452**

Positive selection was evaluated using MEME (*p* ≤ 0.1) and FUBAR (posterior probability ≥ 0.9). A dash (–) indicates that no positively selected site was detected within the corresponding lineage. Positions identified by both MEME and FUBAR are shown in bold. Groups A–E represent phylogenetically defined NA lineages, whereas “Entire dataset” denotes analyses conducted on all sequences combined.

### 3.4 Determination of consensus sequences

Consensus sequences for each phylogenetic group were determined by integrating positive selection signatures with mutation frequency patterns (Table 3).

**TABLE 3:** Consensus sequence construction across NA groups.

Group	Positive selections	Common mutations	Consensus sequence	Adjusted sequences
A	188, 386, 469	V241I, N369K	I188, N200, N386, T452, K469	N/A
B	200, 264, 416, 452	I106V, N200S, V264I, N270K, I314M, I321V, N386K, K432E	S200, V264, N270, K386, D416, T452	B1: V264I, N270K
C	188	I188T, N449D	T188, S200, T452	N/A
D	200, 386, 389	I389K, D416N, T452I	S200, K386, I389, N416, I452	D1: I389K
E	270	N222K, V453M, K469N	S200, K270, I452, V453, K469	E1: V453M, K469N E2: H5N1 integrated

Sites under positive selection in the entire dataset (positions 200 and 452) were conserved across all lineage-specific consensus sequences and therefore were not repeated for each group. Residues identified during selection analyses but lacking sufficient support or functional relevance were excluded from the consensus profiles. “Adjusted sequences” refer to optimized variants that incorporate functionally meaningful substitutions or cross-subtype features (e.g., elements derived from H5N1).

Group-specific consensus sequences were defined after excluding substitutions lacking selection support. In Group A, residues 188, 386, and 469 were under positive selection, forming the A0 consensus together with conserved residues 200 and 452. Group B showed positive selection at residues 200, 264, 416, and 452, resulting in B0 consensus, with an updated B1 variant reflecting V264I and N270K substitutions. Group C exhibited positive selection only at residue 188, yielding the C0 consensus (188, 200, and 452). In Group D, residues 200, 386, and 389 were under positive selection, and two alternative consensus sequences (D0 and D1) were generated based on dominant amino acids at position 389. Because residue 389 harbored multiple coexisting amino acids, early dominance of isoleucine justified its inclusion in the D0 consensus, while lysine at the same site was incorporated into an alternative D1 sequence. Group E exhibited positive selection at residue 270, and additional substitutions at 453 and 469 were incorporated based on their presence in recent isolates. Recent substitutions (V453M and K469N) were incorporated into an adjusted E1 sequence. An extended E2 sequence was developed by incorporating H5N1-specific residues into the seasonal H1N1 backbone to broaden antigenic coverage (Krammer and Schultz-Cherry, 2023). Notably, residue 432 displayed subtype-specific divergence, with lysine in H5N1 versus glutamate in H1N1. This variation was included in E2 to enhance potential cross-protection.

### 3.5 Protective efficacy of NA-based mRNA vaccines

**FIGURE 3:**
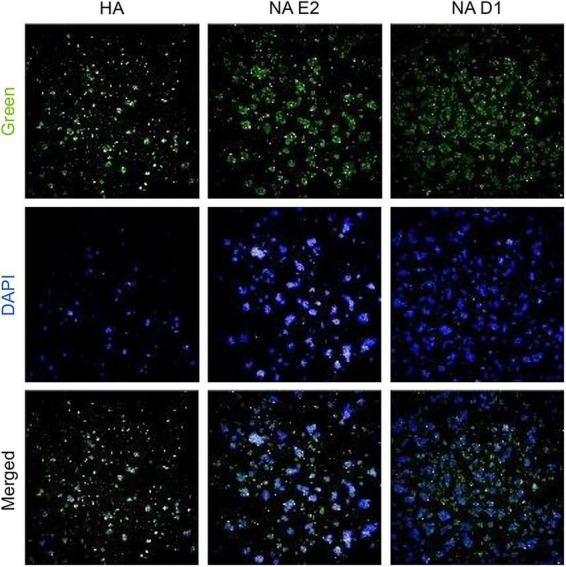
*In vitro* expression of HA and NA mRNA constructs. Immunofluorescence assay (IFA) was performed to evaluate HA and NA protein expression in HEK293T cells transfected with the respective mRNA constructs using Lipofectamine 3,000. The cells were fixed 24 h post-transfection, and HA or NA proteins were detected using NIBSC antisera (21/120, 23/208). Nuclei were counterstained with DAPI to visualize cellular localization.

The mRNA-encoded NA-D1 and NA-E2 antigens were designed using the A/Victoria/4897/2022 (H1N1) strain, which also served as the template for the HA construct. All mRNAs contained a 5′ cap1 structure and N1-methylpseudouridine substitutions to enhance translation and reduce innate immune activation (Easterbrook et al., 2012; Liu et al., 2015; Schulman et al., 1968). Protein expression was confirmed by immunofluorescence assays in HEK293T cells (Figure 3).

**FIGURE 4:**
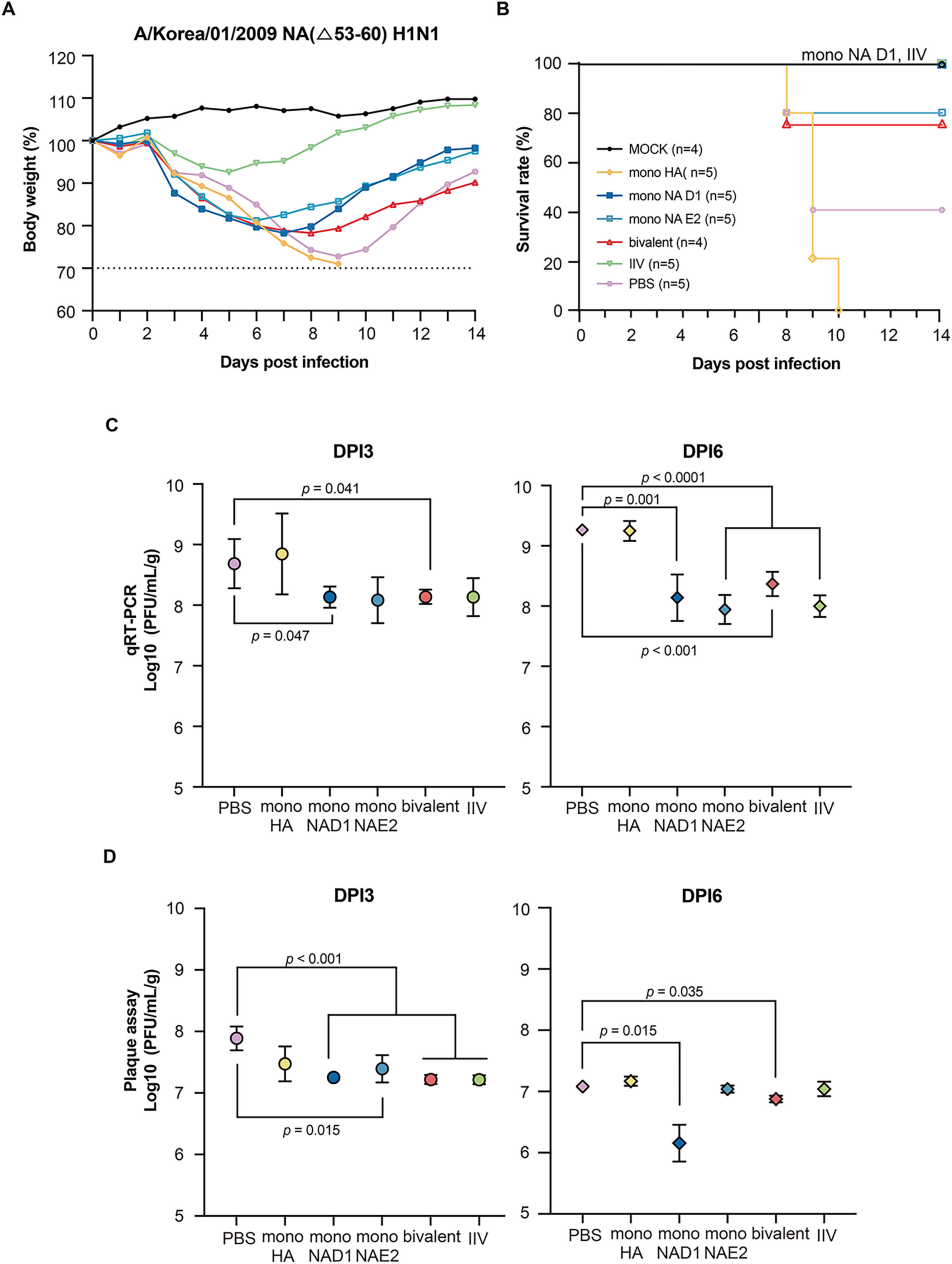
H1N1 influenza virus challenge outcomes in vaccinated mice. **(A)** Body weight changes and **(B)** survival of C57BL/6 mice following challenge with A/Korea/01/2009 (H1N1). Mice were monitored daily for 14 dpi. Four mice from each group were euthanized on 3 and 6 dpi for virological assays. **(C)** Viral RNA levels in lung tissues were quantified by qRT-PCR on 3 and 6 dpi. **(D)** Infectious viral titers in lungs were measured by plaque assay on the same days. Statistical significance was assessed using an unpaired two-tailed Student’s *t*-test.

**FIGURE 5:**
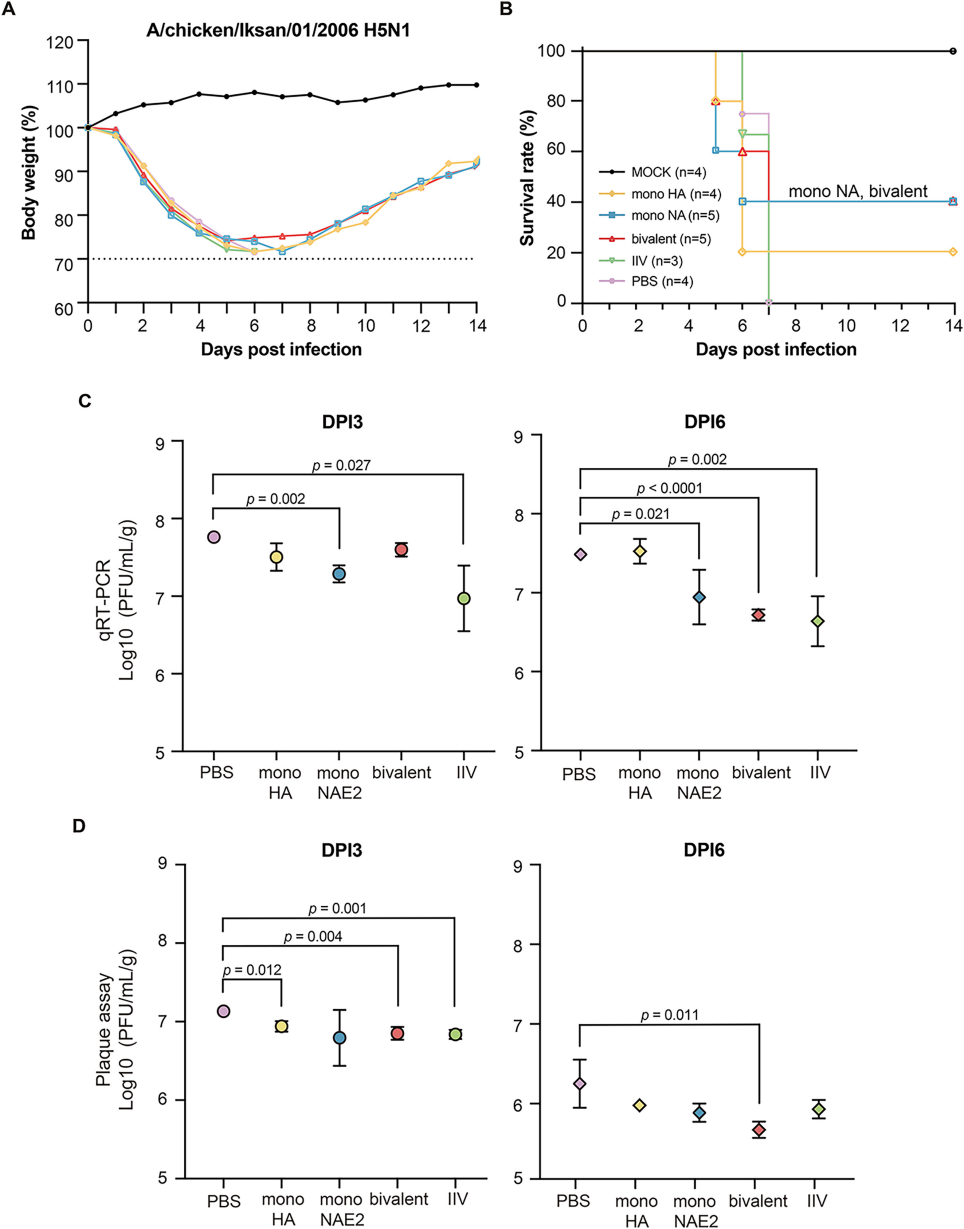
H5N1 influenza virus challenge outcomes in vaccinated mice. **(A)** Body weight changes and **(B)** survival of C57BL/6 mice following challenge with A/chicken/Iksan/01/2006 (H5N1). Mice were monitored daily for 14 dpi. Four mice from each group were euthanized on 3 and 6 dpi for virological assays. **(C)** Viral RNA levels in lung tissues were quantified by qRT-PCR on 3 and 6 dpi. **(D)** Infectious viral titers in lungs were measured by plaque assay on the same days. Statistical significance was assessed using an unpaired two-tailed Student’s *t*-test.

Following homologous H1N1 challenge, mice immunized with mono-NA-D1 or IIV were fully protected, exhibited only transient weight loss followed by rapid recovery. The mono-NA-E2 group showed a similar but slightly more variable pattern. In contrast, PBS and mono-HA immunized mice experienced severe weight loss, resulting in survival rates below 50% (Figures 4A,B). To assess the cross-protection, mice were challenged with 5 MLD_50_ of the heterologous A/chicken/Iksan/01/2006 (H5N1). As expected, IIV- and PBS groups experienced 100% mortality (Figures 5A,B). Partial protection was observed in the bivalent and mono-NA-E2 groups, which achieved survival rate of approximately 40%, whereas mono-HA conferred minimal protection (20% survival). All groups exhibited substantial weight loss following challenge.

Viral replication in the lungs was measured by qRT-qPCR and plaque assays. After homologous H1N1 challenge (Elsharkawy et al., 2025; Kumaki et al., 2011; Lin et al., 2024), viral loads at days post-infection (dpi) 3 in all NA-based mRNA groups were comparable to those of the IIV, whereas mono-HA exhibited significantly higher titers (Figures 4C,D). By dpi 6, plaque assays showed that mono-NA-D1 and the bivalent vaccine maintained the lowest viral titers, consistent with enhanced viral clearance. In the H5N1 challenge model, early viral loads (dpi 3) did not differ significantly among groups, but by dpi 6, the bivalent mRNA vaccine showed the most pronounced reduction in viral titers in both assays, indicating partial heterologous protective activity (Figures 5C,D).

**FIGURE 6:**
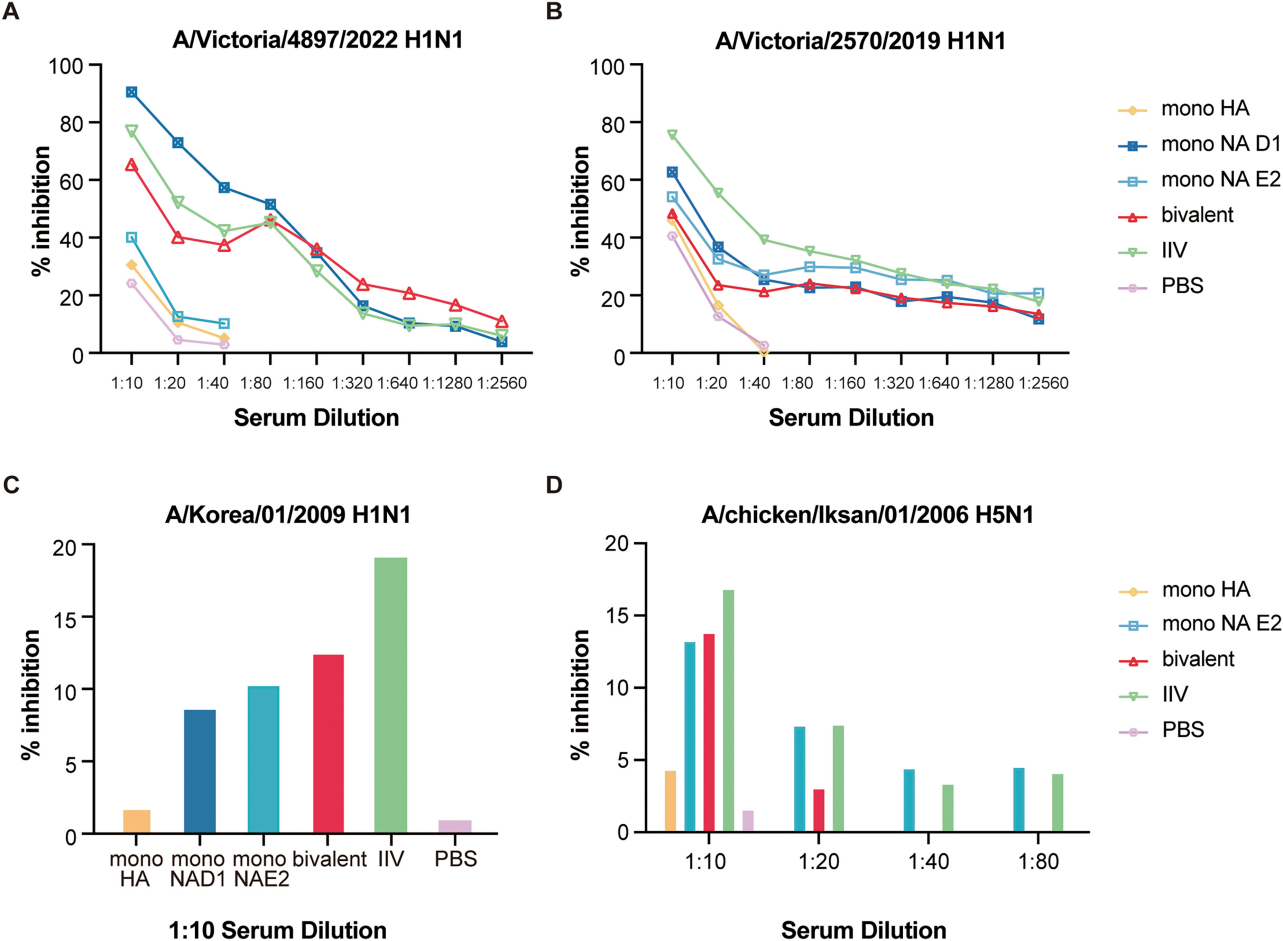
Inhibition of NA enzymatic activity measured by ELLA. Sera collected 27 days after vaccination were serially diluted two-fold and assessed for NI activity using ELLA. NI titers were measured against **(A)** A/Victoria/4897/2022 (H1N1), **(B)** A/Victoria/2570/2019 (H1N1), **(C)** A/Korea/01/2009 (H1N1), and **(D)** A/chicken/Iksan/01/2006 (H5N1).

Immunogenicity was assessed using neuraminidase inhibition (NAI) assays (Couzens et al., 2014). Against the homologous A/Victoria/4897/2022 (H1N1) strain, the mono-NA-D1 vaccine elicited the strongest NAI responses, followed by the bivalent and IIV groups (Figure 6A). Cross-reactive NAI responses were detectable against the heterologous A/Victoria/2570/2019 (H1N1), although IIV induced the highest titers (Figure 6B). Against more antigenically distant viruses (A/Korea/01/2009 and H5N1), NAI activity was uniformly low (< 20%), consistent with the observed partial protection seen *in vivo* (Figures 6C,D).

## 4 Discussion

Seasonal influenza vaccines have traditionally relied on HA as the principal antigenic component (Bouvier and Palese, 2008). However, HA-focused formulations are constrained by extensive antigenic variability, which contributes to mismatch-driven reductions in vaccine effectiveness and hampers preparedness against newly emerging variants (Bliss et al., 2024; Wang et al., 2022). The inflexibility of egg- and cell-based manufacturing systems further restricts timely updates of vaccine strains (Estrada and Schultz-Cherry, 2019; Wang et al., 1989). These limitations highlight the need for vaccine strategies that are less susceptible to antigenic drift and capable of providing more durable breadth of protection. In contrast to HA, NA evolves more slowly and exhibits higher levels of sequence conservation across influenza A viruses (Creytens et al., 2021). NA-inhibiting antibodies can restrict viral egress and mitigate disease severity, even in the absence of robust HA-mediated neutralization (Gilchuk et al., 2019; Tan et al., 2022). Despite these functional advantages, NA remains underrepresented in current vaccines due to HA immunodominance and the lack of standardized NA content (Carascal et al., 2022; Jang and Seong, 2019). These observations underscore NA as a promising yet underexploited antigen for broadening cross-protective immunity.

To identify the targets for NA-centered mRNA vaccine design, we first characterized the evolutionary and structural landscape of post-2009 human H1N1 NA (Figure 1). Bayesian phylogenetic analysis revealed five major NA lineages, each defined by clusters of surface-exposed amino acid substitutions. Structural modeling indicated that most substitutions were located on the NA head domain and positioned away from the catalytic center and tetramerization interfaces (Figure 2), suggesting that these regions may be altered without compromising essential enzymatic features. These observations are consistent with previous reports indicating that NA surface variability can accommodate immune-driven evolution without disrupting core functional constraints (Lei et al., 2023). Analysis of HA-NA reassortment patterns revealed that heterologous segment pairings were relatively infrequent and confined to specific lineage combinations (Table 1), indicating relatively stable HA-NA pairing dynamics. In particular, reassortments were predominantly observed between Groups C and D, whereas Group E maintained complete HA-NA concordance across all subsets analyzed.

Selection analyses further refined antigen design by identifying both conserved and lineage-associated sites with potential relevance for immune engagement (Tables 2, 3). Two positively selected positions 200 and 452 were consistently retained across lineages by both MEME and FUBAR analyses, highlighting their evolutionary significance. These conserved features, combined with structurally compatible recurrent mutations, guided the construction of consensus NA sequences. This integrative process yielded two optimized NA antigens: D1, tailored to preserve H1N1-specific features, and E2, incorporating four lineage-associated H5N1 residues (positions 248, 321, 369, and 432) intended to expand antigenic recognition. Notably, residue 432, a lineage-defining charge-inversion distinguishing human from avian N1 proteins, was included based on its potential to influence antigenic surface properties (Ge et al., 2022). This stepwise workflow enabled selection of mutations that balanced lineage fidelity with broader antigenic coverage.

Both NA-D1 and NA-E2 were robustly expressed *in vitro* (Figure 3), indicating that downstream differences in immunogenicity would likely reflect antigenic rather than expression-related effects. *In vivo*, each construct generated strong NAI responses comparable to those elicited by licensed IIV (Figure 6), confirming that the mRNA-LNP platform effectively presents functional NA. HA-only vaccination produced negligible NI activity, whereas mono-NA-D1 induced the highest inhibition against homologous H1N1, consistent with optimal epitope matching. IIV demonstrated broader reactivity across H1N1 variants, likely reflecting its multicomponent composition. As expected, NAI responses to H5N1 were minimal across all vaccine groups, paralleling the marked antigenic divergence between H1 and H5 lineages. Although the observed NAI confirms functional enzymatic activity, we did not conduct high-resolution structural or biophysical analyses to directly verify conformational equivalence to naive NA. Given prior reports that NA stability and structural presentation in inactivated vaccines can influence immunogenicity (Ellis et al., 2022), comprehensive structural validation would further strengthen translational interpretation.

Challenge experiments further clarified the protective contributions of NA-directed immunity. Against homologous H1N1 challenge, both NA-D1 and NA-E2 conferred protection comparable to IIV, with minimal weight loss and marked reductions in lung viral loads (Figure 4). These results align with prior studies demonstrating that NAI titers correlate with reduced viral replication, decreased shedding, and mitigation of disease severity (Memoli et al., 2016; Momont et al., 2023; Walz et al., 2018). Together, these results support a protective role for NA-specific immunity that is at least partially independent of HA-driven neutralization.

Under heterosubtypic H5N1 challenge conditions, the NA-E2 and bivalent vaccines provided partial protection (Figure 5). Despite sharing only ∼80–83% NA identity with the challenge strain (Table 4), E2 vaccination reduced viral titers at later time points and achieved approximately 40% survival, suggesting a possible contribution of lineage-associated residues to improved cross-lineage recognition. The bivalent formulation exhibited the strongest suppression of viral replication, underscoring the complementary contributions of HA-mediated neutralization and NA-mediated inhibition of viral release. This synergy is consistent with prior reports showing that balanced HA and NA immunity enhances protection against antigenically divergent viruses (Job et al., 2018; Yang et al., 2013).

**TABLE 4:** Comparative nucleotide and amino acid sequence identities of HA and NA mRNA constructs relative to challenge viruses.

Challenge virus	mRNA construct	Nucleotide identity (%)	Amino acid identity (%)
A/chicken/Iksan/01/2006 (H5N1)	HA1	57.558	49.128
	NA-D1	80.581	82.489
	NA-E2	80.865	83.122
A/Korea/01/2009 (H1N1)	HA1	94.767	90.988
	NA-D1	96.809	94.680
	NA-E2	95.887	93.191

Pairwise nucleotide and amino acid sequence identities were calculated between each mRNA construct and corresponding challenge virus strains. Percent identity values represent overall sequence similarity at both the nucleotide and protein levels.

Taken together, our findings support a vaccination strategy in which mRNA vaccine platforms combine strain-specific neutralization from HA with the broader more durable responses generated against NA. Our computational workflow further illustrates how evolutionary and structural analyses can guide rational antigen design can accelerate antigen optimization and enable rapid adaptation to emergent viral threats. Several limitations warrant consideration. The absence of a WHO-recommended H5N1 vaccine comparator limited a full assessment of cross-protective breadth. Moreover, NA-based constructs conferred only partial protection against highly divergent subtypes, indicating that broader cross-protection may require incorporation of highly conserved epitopes or implementation of polyvalent antigen designs. In this study, NA antigens were deliberately evaluated independently of HA to isolate NA-specific immunogenic effects and minimize HA-driven immunodominance. However, it does not fully capture the complex interplay between HA and NA. Co-optimization strategies may improve balanced immune induction and reduce inter-antigen interference in multivalent mRNA formulations. Importantly, the mouse model used in this study does not fully recapitulate the complexity of human immunity. In human, pre-existing immunity and immune imprinting can profoundly influence epitope targeting, antibody hierarchy, and functional antibody profiles not reflected in immunologically naïve animals. Furthermore, NA-mediated protection is not solely attributable to neuraminidase inhibition. Fc-dependent effector mechanisms, including antibody-dependent cellular cytotoxicity, and antibody-dependent cellular phagocytosis, and complement activation, may also contribute to *in vivo* protection but were not directly assessed in the present study.

Evolutionary sequence conservation does not necessarily equate to antigenic conservation, and computational predictions cannot fully capture the complexity of antigen-antibody interaction. This distinction was evident in the reduced inhibition observed against A/Korea/01/2009 (H1N1) despite high NA sequence identity, suggesting that limited amino acid substitutions may affect functional inhibition. Integrating antigenicity-informed mapping with iterative experimental validation by incorporating quantitative NAI titers and strain-specific inhibition data into residue-level modeling could refine predictive accuracy and enable progressive antigen optimization.

In summary, computationally guided NA-based mRNA vaccines conferred robust protection against homologous H1N1 challenge and partial protection against heterosubtypic H5N1 challenge, with efficacy patterns reflecting underlying antigenic relatedness. These results highlight NA’s value as a complementary antigen and establish a proof-of-concept framework for integrating evolutionary analysis with mRNA platforms to advance universal influenza development.

## Data Availability

The original contributions presented in the study are included in the article/Supplementary material, further inquiries can be directed to the corresponding author.

## Footnotes

1
https://www.ncbi.nlm.nih.gov

2
http://www.gisaid.org

## Supplementary material

Supplementary Figure 1 Experimental design for mouse vaccination and viral challenge. Female C57BL/6 mice were intramuscularly vaccinated at 2-week intervals. Serum samples were collected on day 27. On day 28, mice were intranasally challenged with mouse-adapted A/Korea/01/2009 (H1N1) or A/chicken/Iksan/01/2006 (H5N1) virus. For lung viral titration, four mice per group were euthanized at 3 and 6 days post-infection. The remaining mice were monitored daily for body weight loss and survival for 14 days post-challenge. The group allocation shown in this schematic corresponds to the H1N1 challenge.

Supplementary Figure 2 Reassortment patterns between HA and NA genes in H1N1 viruses. **(A)** tanglegram depicts the phylogenetic relationships of HA (left) and NA (right) genes from H1N1 viruses. Viruses are grouped into genetic clusters (A–E), with representative samples randomly selected (**A:** random 1; **B:** random 2; **C:** random 3). Colored lines link HA-NA pairs from the same virus, where crossing lines denote reassortment events. Shared amino acid mutations are labeled at internal nodes, with lysine mutations shown in red. The *x*-axes indicate the number of mutations (left) and genetic divergence (right).

## References

[B1] Anderson L. Hoyt C. Zucker J. McNaughton A. Teuton J. Karis K. et al. (2025). Computational tools and data integration to accelerate vaccine development: Challenges, opportunities, and future directions. *Front. Immunol.* 16:1502484. 10.3389/fimmu.2025.1502484 40124369 PMC11925797

[B2] Arevalo C. Bolton M. Le Sage V. Ye N. Furey C. Muramatsu H. et al. (2022). A multivalent nucleoside-modified mRNA vaccine against all known influenza virus subtypes. *Science* 378 899–904. 10.1126/science.abm0271 36423275 PMC10790309

[B3] Bliss C. Nachbagauer R. Mariottini C. Cuevas F. Feser J. Naficy A. et al. (2024). A chimeric haemagglutinin-based universal influenza virus vaccine boosts human cellular immune responses directed towards the conserved haemagglutinin stalk domain and the viral nucleoprotein. *EBioMedicine* 104:105153. 10.1016/j.ebiom.2024.105153 38805853 PMC11154122

[B4] Bouvier N. M. Palese P. (2008). The biology of influenza viruses. *Vaccine* 26 D49–D53. 10.1016/j.vaccine.2008.07.039 19230160 PMC3074182

[B5] Brochut M. Heinonen T. Snäkä T. Gilbert C. Le Roy D. Roger T. (2024). Using weight loss to predict outcome and define a humane endpoint in preclinical sepsis studies. *Sci. Rep.* 14:21150. 10.1038/s41598-024-72039-1 39256525 PMC11387420

[B6] Carascal M. B. Pavon R. D. N. Rivera W. L. (2022). Recent progress in recombinant influenza vaccine development toward heterosubtypic immune response. *Front. Immunol.* 13:878943. 10.3389/fimmu.2022.878943 35663997 PMC9162156

[B7] Choi E. Kim H. Baek Y. Kim E. Pascua P. Park S. et al. (2014). Differential microRNA expression following infection with a mouse-adapted, highly virulent avian H5N2 virus. *BMC Microbiol.* 14:252. 10.1186/s12866-014-0252-0 25266911 PMC4189662

[B8] Choi Y. Song J. Wie S. Choi W. Lee J. Lee J. et al. (2024). Real-world effectiveness of influenza vaccine over a decade during the 2011-2021 seasons-Implications of vaccine mismatch. *Vaccine* 42:126381. 10.1016/j.vaccine.2024.126381 39362009

[B9] Couzens L. Gao J. Westgeest K. Sandbulte M. Lugovtsev V. Fouchier R. et al. (2014). An optimized enzyme-linked lectin assay to measure influenza A virus neuraminidase inhibition antibody titers in human sera. *J. Virol. Methods* 210 7–14. 10.1016/j.jviromet.2014.09.003 25233882

[B10] Creytens S. Pascha M. Ballegeer M. Saelens X. de Haan C. (2021). Influenza neuraminidase characteristics and potential as a vaccine target. *Front. Immunol.* 12:786617. 10.3389/fimmu.2021.786617 34868073 PMC8635103

[B11] Easterbrook J. Schwartzman L. Gao J. Kash J. Morens D. Couzens L. et al. (2012). Protection against a lethal H5N1 influenza challenge by intranasal immunization with virus-like particles containing 2009 pandemic H1N1 neuraminidase in mice. *Virology* 432 39–44. 10.1016/j.virol.2012.06.003 22727831 PMC3725556

[B12] Eichelberger M. Morens D. Taubenberger J. (2018). Neuraminidase as an influenza vaccine antigen: A low hanging fruit, ready for picking to improve vaccine effectiveness. *Curr. Opin. Immunol.* 53 38–44. 10.1016/j.coi.2018.03.025 29674167 PMC6141346

[B13] Ellis D. Lederhofer J. Acton O. Tsybovsky Y. Kephart S. Yap C. et al. (2022). Structure-based design of stabilized recombinant influenza neuraminidase tetramers. *Nat. Commun.* 13:1825. 10.1038/s41467-022-29416-z 35383176 PMC8983682

[B14] Elsharkawy A. Jahantigh H. Guglani A. Stone S. Arora K. Kumar M. (2025). Virus-specific host responses and gene signatures following infection with major SARS-CoV-2 variants of concern: Role of ZBP1 in viral clearance and lung inflammation. *Front. Immunol.* 16:1557535. 10.3389/fimmu.2025.1557535 40416961 PMC12098559

[B15] Estrada L. Schultz-Cherry S. (2019). Development of a universal influenza vaccine. *J. Immunol.* 202 392–398. 10.4049/jimmunol.1801054 30617121 PMC6327971

[B16] Ge J. Lin X. Guo J. Liu L. Li Z. Lan Y. et al. (2022). The antibody response against neuraminidase in human influenza A (H3N2) virus infections during 2018/2019 flu season: Focusing on the epitopes of 329- N-glycosylation and E344 in N2. *Front. Microbiol.* 13:845088. 10.3389/fmicb.2022.845088 35387078 PMC8978628

[B17] Gilchuk I. Bangaru S. Gilchuk P. Irving R. Kose N. Bombardi R. et al. (2019). Influenza H7N9 virus neuraminidase-specific human monoclonal antibodies inhibit viral egress and protect from lethal influenza infection in mice. *Cell Host Microbe* 26 715–728.e8. 10.1016/j.chom.2019.10.003 31757769 PMC6941661

[B18] Gravel C. Li C. Wang J. Hashem A. Jaentschke B. Xu K. et al. (2010). Qualitative and quantitative analyses of virtually all subtypes of influenza A and B viral neuraminidases using antibodies targeting the universally conserved sequences. *Vaccine* 28 5774–5784. 10.1016/j.vaccine.2010.06.075 20621113

[B19] Henderson J. Ujita A. Hill E. Yousif-Rosales S. Smith C. Ko N. et al. (2021). Cap 1 messenger RNA synthesis with co-transcriptional CleanCap^®^ analog by in vitro transcription. *Curr. Protoc.* 1:e39. 10.1002/cpz1.39 33524237

[B20] Iuliano A. Roguski K. Chang H. Muscatello D. Palekar R. Tempia S. et al. (2018). Estimates of global seasonal influenza-associated respiratory mortality: A modelling study. *Lancet* 391 1285–1300. 10.1016/S0140-6736(17)33293-2 29248255 PMC5935243

[B21] Jang Y. Seong B. (2019). The quest for a truly universal influenza vaccine. *Front. Cell. Infect. Microbiol.* 9:344. 10.3389/fcimb.2019.00344 31649895 PMC6795694

[B22] Job E. Ysenbaert T. Smet A. Christopoulou I. Strugnell T. Oloo E. et al. (2018). Broadened immunity against influenza by vaccination with computationally designed influenza virus N1 neuraminidase constructs. *NPJ Vaccines* 3:55. 10.1038/s41541-018-0093-1 30510776 PMC6265323

[B23] Katoh K. Standley D. M. (2013). MAFFT multiple sequence alignment software version 7: Improvements in performance and usability. *Mol. Biol. Evol.* 30 772–780. 10.1093/molbev/mst010 23329690 PMC3603318

[B24] Kauffman K. Mir F. Jhunjhunwala S. Kaczmarek J. Hurtado J. Yang J. et al. (2016). Efficacy and immunogenicity of unmodified and pseudouridine-modified mRNA delivered systemically with lipid nanoparticles in vivo. *Biomaterials* 109 78–87. 10.1016/j.biomaterials.2016.09.006 27680591 PMC5267554

[B25] Kim P. Jang Y. Kwon S. Lee C. Han G. Seong B. (2018). Glycosylation of hemagglutinin and neuraminidase of influenza A Virus as signature for ecological spillover and adaptation among influenza reservoirs. *Viruses* 10:183. 10.3390/v10040183 29642453 PMC5923477

[B26] Krammer F. Schultz-Cherry S. (2023). We need to keep an eye on avian influenza. *Nat. Rev. Immunol.* 23 267–268. 10.1038/s41577-023-00868-8 36944755 PMC10028763

[B27] Kumaki Y. Wandersee M. Smith A. Zhou Y. Simmons G. Nelson N. et al. (2011). Inhibition of severe acute respiratory syndrome coronavirus replication in a lethal SARS-CoV BALB/c mouse model by stinging nettle lectin, Urtica dioica agglutinin. *Antiviral Res.* 90 22–32. 10.1016/j.antiviral.2011.02.003 21338626 PMC3085190

[B28] Lei R. Hernandez Garcia A. Tan T. Teo Q. Wang Y. Zhang X. et al. (2023). Mutational fitness landscape of human influenza H3N2 neuraminidase. *Cell. Rep.* 42:111951. 10.1016/j.celrep.2022.111951 36640354 PMC9931530

[B29] Leonard R. Burke K. Spreng R. Macintyre A. Tam Y. Alameh M. et al. (2024). Improved influenza vaccine responses after expression of multiple viral glycoproteins from a single mRNA. *Nat. Commun.* 15:8712. 10.1038/s41467-024-52940-z 39379405 PMC11461824

[B30] Li W. Godzik A. (2006). Cd-hit: A fast program for clustering and comparing large sets of protein or nucleotide sequences. *Bioinformatics* 22 1658–1659. 10.1093/bioinformatics/btl158 16731699

[B31] Li Y. Wang X. Zeng X. Ren W. Liao P. Zhu B. (2023). Protective efficacy of a universal influenza mRNA vaccine against the challenge of H1 and H5 influenza A viruses in mice. *mLife* 2 308–316. 10.1002/mlf2.12085 38817814 PMC10989953

[B32] Lin Y. Khan M. Weynand B. Laporte M. Coenjaerts F. Babusis D. et al. (2024). A robust mouse model of HPIV-3 infection and efficacy of GS-441524 against virus-induced lung pathology. *Nat. Commun.* 15:7765. 10.1038/s41467-024-52071-5 39237507 PMC11377736

[B33] Liu J. Xia Y. Tian C. Chen Z. Guo W. Liu Y. et al. (2025). E2-based mRNA vaccine encapsulated in lipid nanoparticles protects pigs against classical swine fever virus. *J. Virol.* 99:e0097825. 10.1128/jvi.00978-25 40838721 PMC12456140

[B34] Liu W. Lin C. Tsou Y. Jan J. Wu S. (2015). Cross-reactive neuraminidase-inhibiting antibodies elicited by immunization with recombinant neuraminidase proteins of H5N1 and pandemic H1N1 influenza A viruses. *J. Virol.* 89 7224–7234. 10.1128/JVI.00585-15 25948745 PMC4473581

[B35] Liu Y. Strohmeier S. González-Domínguez I. Tan J. Simon V. Krammer F. et al. (2021). Mosaic hemagglutinin-based whole inactivated virus vaccines induce broad protection against influenza B virus challenge in mice. *Front. Immunol.* 12:746447. 10.3389/fimmu.2021.746447 34603333 PMC8481571

[B36] McAuley J. Gilbertson B. Trifkovic S. Brown L. McKimm-Breschkin J. (2019). Influenza virus neuraminidase structure and functions. *Front. Microbiol.* 10:39. 10.3389/fmicb.2019.00039 30761095 PMC6362415

[B37] Memoli M. Shaw P. Han A. Czajkowski L. Reed S. Athota R. et al. (2016). Evaluation of antihemagglutinin and antineuraminidase antibodies as correlates of protection in an influenza A/H1N1 virus healthy human challenge model. *mBio* 7:e00417-16. 10.1128/mBio.00417-16 27094330 PMC4959521

[B38] Momont C. Dang H. Zatta F. Hauser K. Wang C. di Iulio J. et al. (2023). A pan-influenza antibody inhibiting neuraminidase via receptor mimicry. *Nature* 618 590–597. 10.1038/s41586-023-06136-y 37258672 PMC10266979

[B39] Mortazavi M. Pirbonyeh N. Javanmardi F. Emami A. (2023). Bioinformatics and structural analysis of antigenic variation in the hemagglutinin gene of the influenza A(H1N1)pdm09 virus circulating in Shiraz (2013 to 2015). *Microbiol. Spectr.* 11:e0463022. 10.1128/spectrum.04630-22 37436149 PMC10433955

[B40] Nachbagauer R. Feser J. Naficy A. Bernstein D. Guptill J. Walter E. et al. (2021). A chimeric hemagglutinin-based universal influenza virus vaccine approach induces broad and long-lasting immunity in a randomized, placebo-controlled phase I trial. *Nat. Med.* 27 106–114. 10.1038/s41591-020-1118-7 33288923

[B41] Schulman J. Khakpour M. Kilbourne E. D. (1968). Protective effects of specific immunity to viral neuraminidase on influenza virus infection of mice. *J. Virol.* 2 778–786. 10.1128/JVI.2.8.778-786.1968 5701819 PMC375691

[B42] Shu Y. McCauley J. (2017). GISAID: Global initiative on sharing all influenza data - from vision to reality. *Euro Surveill.* 22:30494. 10.2807/1560-7917.ES.2017.22.13.30494 28382917 PMC5388101

[B43] Skarlupka A. Bebin-Blackwell A. Sumner S. Ross T. (2021). Universal influenza virus neuraminidase vaccine elicits protective immune responses against human seasonal and pre-pandemic strains. *J. Virol.* 95:e0075921. 10.1128/JVI.00759-21 34160258 PMC8354223

[B44] Stachyra A. Redkiewicz P. Kosson P. Protasiuk A. Góra-Sochacka A. Kudla G. et al. (2016). Codon optimization of antigen coding sequences improves the immune potential of DNA vaccines against avian influenza virus H5N1 in mice and chickens. *Virol. J.* 13:143. 10.1186/s12985-016-0599-y 27562235 PMC5000471

[B45] Suchard M. Lemey P. Baele G. Ayres D. Drummond A. Rambaut A. (2018). Bayesian phylogenetic and phylodynamic data integration using BEAST 1.10. *Virus Evol.* 4:vey016. 10.1093/ve/vey016 29942656 PMC6007674

[B46] Tan J. O’Dell G. Hernandez M. Sordillo E. Kahn Z. Kriti D. et al. (2022). Human anti-neuraminidase antibodies reduce airborne transmission of clinical influenza virus isolates in the guinea pig model. *J. Virol.* 96:e0142121. 10.1128/JVI.01421-21 34669506 PMC8791283

[B47] Tian Y. Deng Z. Chuai Z. Li C. Chang L. Sun F. et al. (2024). A combination influenza mRNA vaccine candidate provided broad protection against diverse influenza virus challenge. *Virology* 596:110125. 10.1016/j.virol.2024.110125 38805804

[B48] Walz L. Kays S. Zimmer G. von Messling V. (2018). Neuraminidase-inhibiting antibody titers correlate with protection from heterologous influenza virus strains of the same neuraminidase subtype. *J. Virol.* 92:e01006-18. 10.1128/JVI.01006-18 29925654 PMC6096819

[B49] Wang M. Katz J. Webster R. (1989). Extensive heterogeneity in the hemagglutinin of egg-grown influenza viruses from different patients. *Virology* 171 275–279. 10.1016/0042-6822(89)90538-2 2741346

[B50] Wang W. Sayedahmed E. Sambhara S. Mittal S. (2022). Progress towards the development of a universal influenza vaccine. *Viruses* 14:1684. 10.3390/v14081684 36016306 PMC9415875

[B51] Wilson J. Guo Z. Reber A. Kamal R. Music N. Gansebom S. et al. (2016). An influenza A virus (H7N9) anti-neuraminidase monoclonal antibody with prophylactic and therapeutic activity in vivo. *Antiviral Res.* 135 48–55. 10.1016/j.antiviral.2016.10.001 27713074 PMC5729279

[B52] Xie H. Wan X. Ye Z. Plant E. Zhao Y. Xu Y. et al. (2015). H3N2 mismatch of 2014-15 Northern hemisphere influenza vaccines and head-to-head comparison between human and ferret antisera derived antigenic maps. *Sci. Rep.* 5:15279. 10.1038/srep15279 26472175 PMC4607887

[B53] Yang C. Skiena S. Futcher B. Mueller S. Wimmer E. (2013). Deliberate reduction of hemagglutinin and neuraminidase expression of influenza virus leads to an ultraprotective live vaccine in mice. *Proc. Natl. Acad. Sci. U S A.* 110 9481–9486. 10.1073/pnas.1307473110 23690603 PMC3677463

[B54] Zhang T. Yin C. Boyd D. Quarato G. Ingram J. Shubina M. et al. (2020). Influenza virus Z-RNAs induce ZBP1-mediated necroptosis. *Cell* 180 1115–1129.e13. 10.1016/j.cell.2020.02.050 32200799 PMC7153753

[B55] Zhou L. Wubshet A. Zhang J. Hou S. Yao K. Zhao Q. et al. (2024). The mRNA vaccine expressing single and fused structural proteins of porcine reproductive and respiratory syndrome induces strong cellular and humoral immune responses in BalB/C Mice. *Viruses* 16:544. 10.3390/v16040544 38675887 PMC11054013

