# Correction: Cross-protective efficacy of NA-based mRNA vaccine candidates against seasonal and avian influenza viruses

**DOI:** 10.3389/fmicb.2026.1855224

**Published:** 2026-04-27

**Authors:** Hyunbeen Kim, Sangyi Lee, Seunghye Cho, Woojin Shin, Soyoung Lee, Sejik Park, Atanas V. Demirev, Taeyoung Lee, You-Jin Kim, Dokeun Kim, Sae Am Song, Kyung Ran Jun, Jin Il Kim

**Affiliations:** 1 Department of Microbiology, Institute for Viral Diseases, Korea University College of Medicine, Seoul, Republic of Korea; 2 Division of Infectious Diseases Vaccine Research, Center for Vaccine Research, National Institute of Infectious Diseases, Korea National Institute of Health, Osong, Republic of Korea; 3 Department of Lab Medicine, Haeundae Paik Hospital, Inje University College of Medicine, Busan, Republic of Korea; 4 Vaccine Innovation Center, Korea University College of Medicine, Seoul, Republic of Korea; 5 Biosafety Center, Korea University College of Medicine, Seoul, Republic of Korea

**Keywords:** hemagglutinin, influenza, mRNA vaccine, neuraminidase, viral variant

An incorrect number was provided for Korea Disease Control and Prevention Agency. The correct number is [2024-ER2407-00].

The original version of this article has been updated.

