# Supplementary figures and images for: Cross-protective efficacy of NA-based mRNA vaccine candidates against seasonal and avian influenza viruses
Source: Front Microbiol. 2026 Apr 9;17:1791088. doi: 10.3389/fmicb.2026.1791088 (PMC13102672; doi:10.3389/fmicb.2026.1791088)

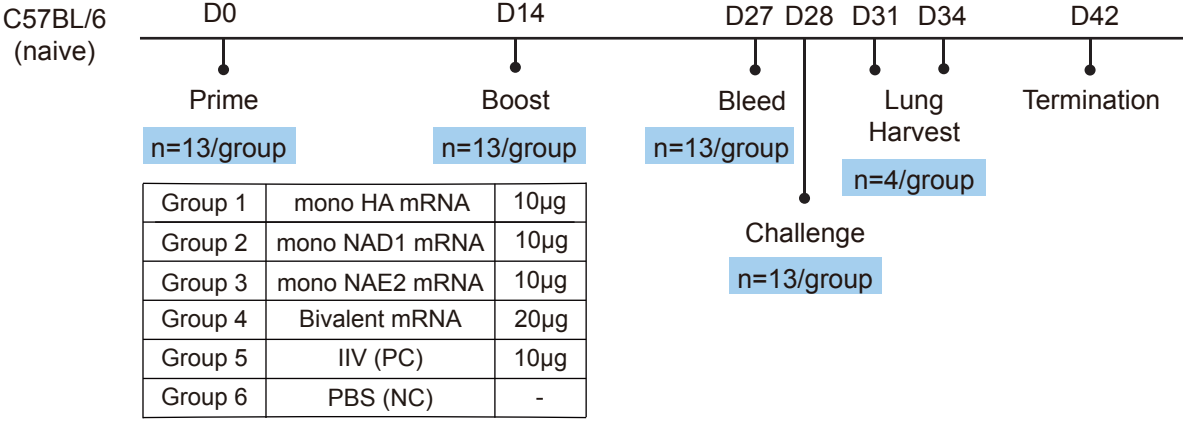

Supplement: Supplementary Figure 1 — Experimental design for mouse vaccination and viral challenge. Female C57BL/6 mice were intramuscularly vaccinated at 2-week intervals. Serum samples were collected on day 27. On day 28, mice were intranasally challenged with mouse-adapted A/Korea/01/2009 (H1N1) or A/chicken/Iksan/01/2006 (H5N1) virus. For lung viral titration, four mice per group were euthanized at 3 and 6 days post-infection. The remaining mice were monitored daily for body weight loss and survival for 14 days post-challenge. The group allocation shown in this schematic corresponds to the H1N1 challenge. [file Data_Sheet_1.pdf]

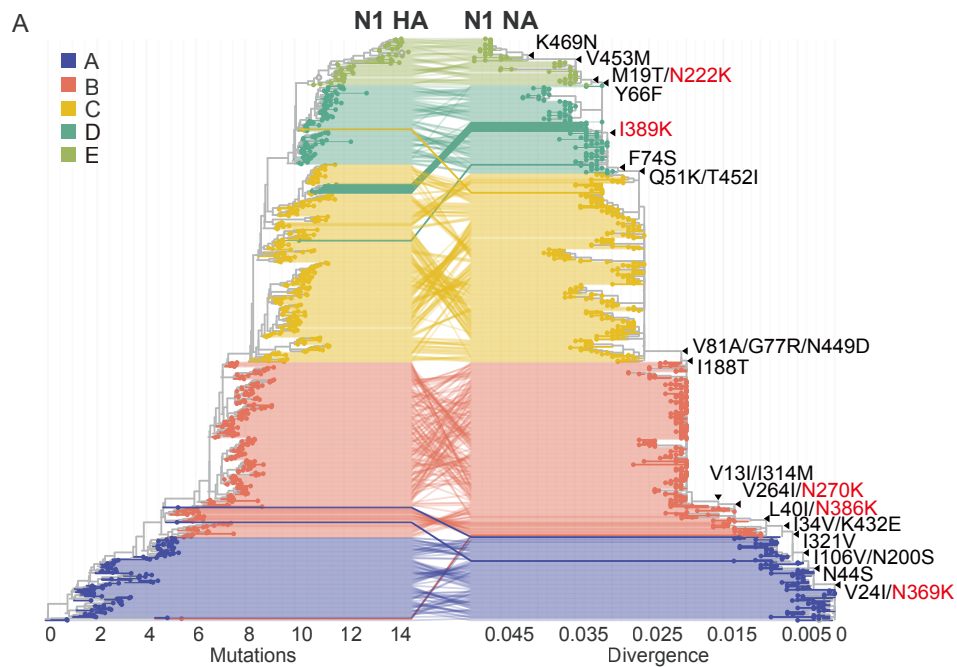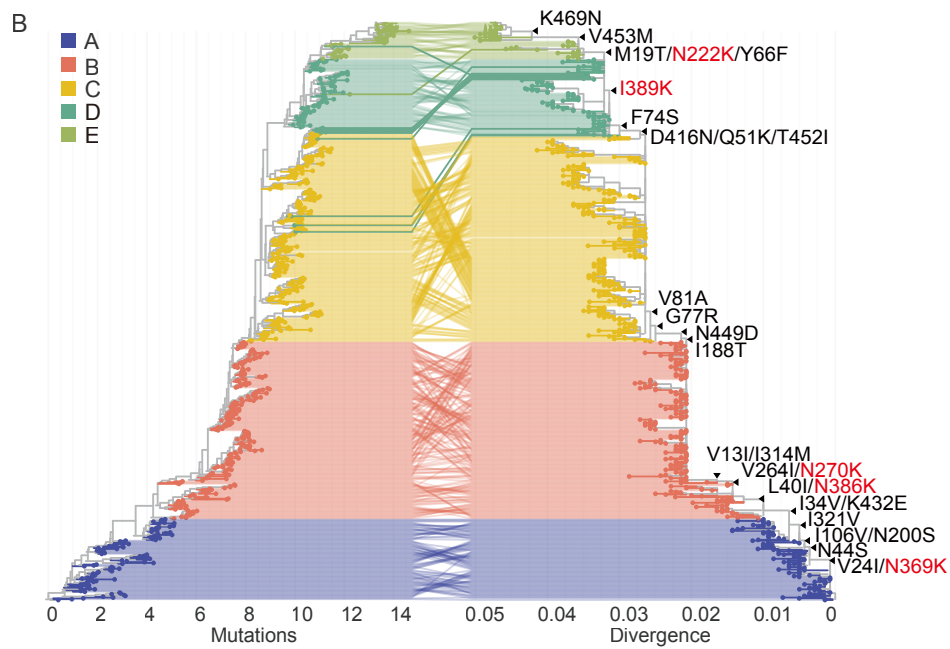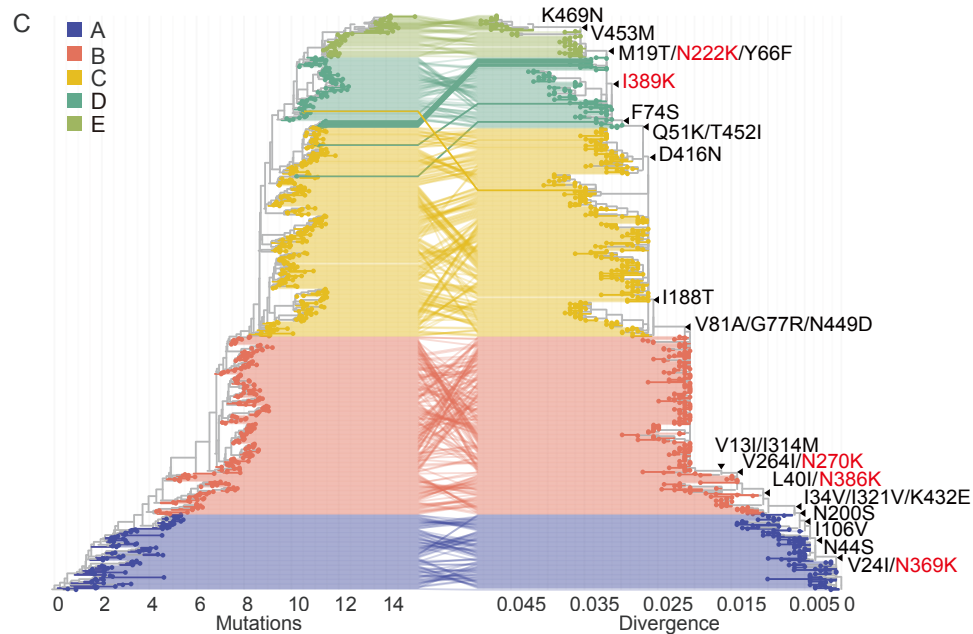

Supplement: Supplementary Figure 2 — Reassortment patterns between HA and NA genes in H1N1 viruses. (A) tanglegram depicts the phylogenetic relationships of HA (left) and NA (right) genes from H1N1 viruses. Viruses are grouped into genetic clusters (A–E), with representative samples randomly selected (A: random 1; B: random 2; C: random 3). Colored lines link HA-NA pairs from the same virus, where crossing lines denote reassortment events. Shared amino acid mutations are labeled at internal nodes, with lysine mutations shown in red. The x-axes indicate the number of mutations (left) and genetic divergence (right). [file Data_Sheet_2.pdf]
